# Influences of anthropogenic land use on microbial community structure and functional potentials of stream benthic biofilms

**DOI:** 10.1038/s41598-017-15624-x

**Published:** 2017-11-08

**Authors:** Xiaodong Qu, Ze Ren, Haiping Zhang, Min Zhang, Yuhang Zhang, Xiaobo Liu, Wenqi Peng

**Affiliations:** 1 Department of Water Environment, China Institute of Water Resources and Hydropower Research, Beijing, 100038 China; 2 State Key Laboratory of Simulation and Regulation of Water Cycle in River Basin, China Institute of Water Resources and Hydropower Research, Beijing, 100038 China; 3 Flathead Lake Biological Station, University of Montana, Polson, MT 59860 USA; 4 College of Water Conservancy, Shenyang Agricultural University, Shenyang, 110866 China

## Abstract

Stream ecosystems are the primary receivers of nutrient and organic carbon exported from terrestrial ecosystems and are profoundly influenced by the land use of the surrounding landscape. The aquatic impacts of anthropogenic land use are often first observed in stream benthic biofilms. We studied the benthic biofilms in streams flowing through forest (upstream) and anthropogenic land use (downstream) areas in southwestern China. The results showed that anthropogenic land use increased nutrient and organic carbon in both stream water and benthic biofilms, which are closely related to the differences in the microbial communities. The taxonomic dissimilarity of the communities was significantly correlated with the functional gene dissimilarity, and the upstream sites had more distinct functional genes. Network analysis showed that upstream sites had more highly connected microbial networks. Furthermore, downstream sites had higher relative abundances of anammox and denitrification suggesting stronger nitrogen removal than upstream sites. Increased nutrients in both the stream water and biofilms caused by anthropogenic land use had severe impacts on the nitrogen cycle in stream ecosystems. Downstream sites also had stronger carbon metabolism than upstream sites. This study provides insights into the influences of anthropogenic land use on microbial community structure and functions of stream benthic biofilms.

## Introduction

Natural landscapes are transformed by anthropogenic activities to satisfy human needs through processes such as agriculture and urbanization^1^. Aquatic ecosystems are sentinels and integrators of terrestrial processes because they are closely related to changes in terrestrial landscapes through the transport and storage of water, nutrients, and energy^2^. As the primary receivers of nutrient and organic matter exported from terrestrial ecosystems, stream ecosystems are profoundly influenced by the land use of their surrounding landscapes^3–5^. In streams heavily influenced by agricultural and urban land uses, elevated nutrient and organic matter concentrations are expected^6–8^.

The aquatic impacts of land use can often first be observed in their effects on stream benthic biofilms^9,10^, which are likely linked to watershed conditions because land use significantly controls the export of carbon (C), nitrogen (N), and phosphorus (P) from catchments to aquatic ecosystems^11–13^, potentially shifting the *in situ* microbial community structure and bacteria mediated processes. Previous studies have demonstrated the variations in stream benthic algae composition in relation to landcover change from forest to agriculture to urban areas^14–16^, close relationships between longitudinal patterns of stream biofilm biomass and pasture degradation^17^, and an influence of landcover conditions on biofilm stoichiometry^18^.

In streams, biofilms are hot spots of microbial activity^19^, contributing substantially to the metabolism and biogeochemical cycles through nutrient uptake, transfer of nutrients to higher trophic levels, and remineralization^20–23^. Various heterotrophic and autotrophic taxa are tightly linked to each other via the transfer of various types of organic matters and nutrient associated with different biological processes^24–28^. Catchment land use significantly controls the export of organic matter and nutrient from catchments to lake ecosystems through streams and rivers and ultimately impacts the suitability of water resources^11–13^. In particular, the nitrogen metabolism in streams is one of the most important ecosystem functions to remove nitrogen^24^ and consequently protect downstream lakes from eutrophication. Moreover, bacteria can account for a substantial portion of the transformation and use of organic matter in riverine systems^29,30^, with implications for ecosystem processes, food webs, and greenhouse gases^31^. Thus, the major metabolisms in riverine systems are of importance because of the potential for riverine ecosystems to store, mineralize, and transport organic matter and nutrient before reaching the downstream aquatic ecosystems^32–34^. Considerable work focused on export, source, transport, retention, and metabolism of nitrogen and carbon in streams that are impacted by anthropogenic activities^27,31,34,35^. However, the influences of anthropogenic land use on microbial community structures and functional potentials of stream biofilms are remain unclear.

Aquatic ecosystems face increasing pressures and threats from disturbances of watersheds associated with land use^36,37^. Understanding the microbial community and the major metabolic pathways is crucial to obtain insights into ecosystem structures and processes^38,39^. In this work, we studied the benthic biofilms in streams flowing through forests and anthropogenic land use (agriculture and urban) areas in the Erhai Lake watershed in southwestern China. The microbial communities of benthic biofilms in upstream and downstream of anthropogenic land use area were compared. We determined the bacterial communities using high-throughput 16S rRNA gene sequencing and investigated carbon and nitrogen metabolic pathways from the PICRUSt (Phylogenetic Investigation of Communities by Reconstruction of Unobserved STates) predicted functional genes encoding the enzymes required in carbon and nitrogen metabolic pathways. Although metagenomics only reflects a potential rather than a realized functional capacity, our data offered an approach to understand and discuss the anthropogenic influences on the microbial community structures and functions in stream benthic biofilms. We hypothesized that (1) anthropogenic land use leads to nutrient and organic carbon increase and alters microbial community structure, and (2) anthropogenic land use alters the nitrogen and carbon metabolic pathways, which are associated with the community structure and nutrient concentrations.

## Methods

### Study area

**Figure 1:**
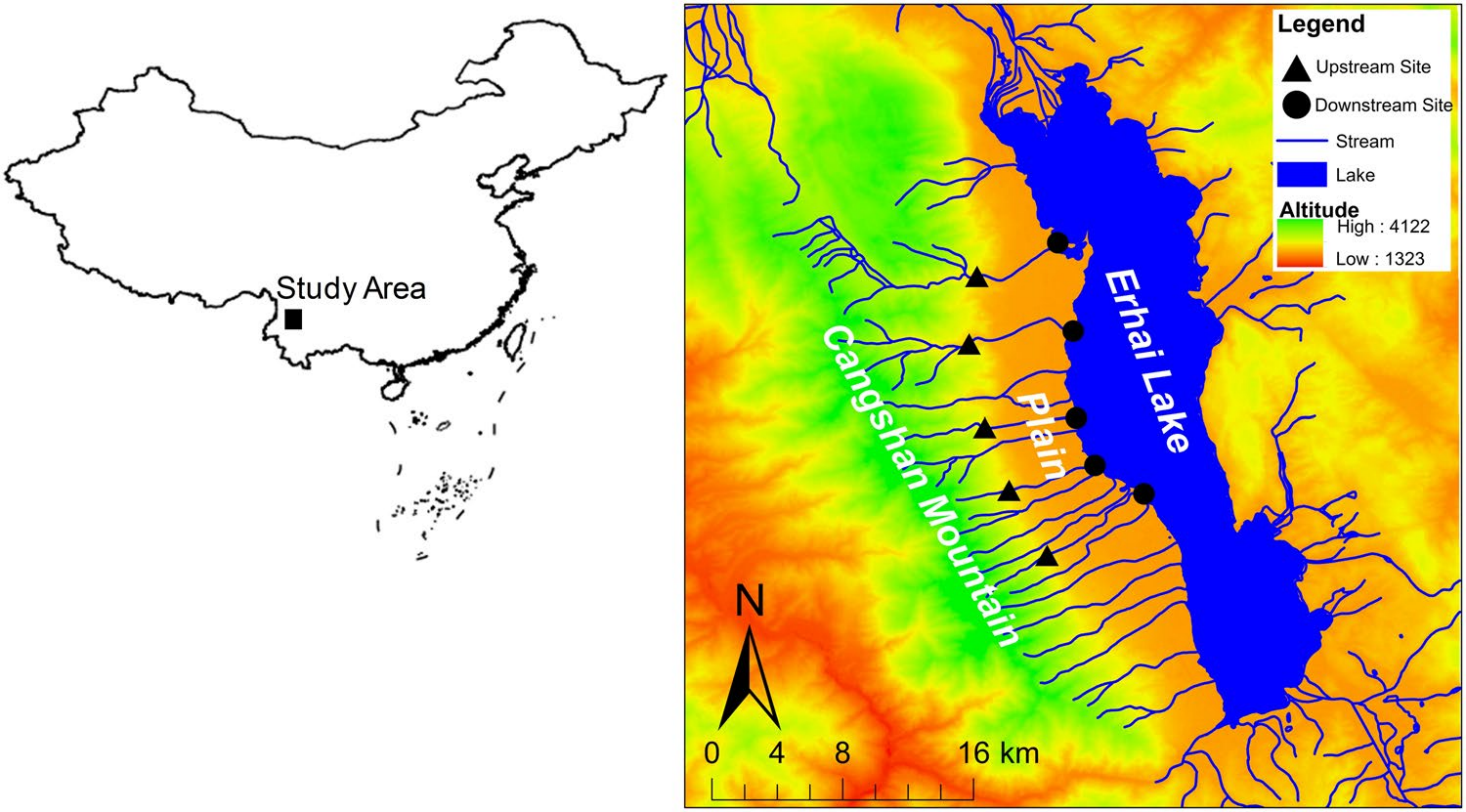
Study area and sample sites. The map was created in ArcGIS 14.0 (http://desktop.arcgis.com/en/arcmap/) using ASTER GDEM data download from USGS (ASTER GDEM is a product of METI and NASA).

The study streams are located on Cangshan Mountain, Yunnan Province, southwestern China (Fig. 1). Cangshan Mountain has 19 peaks. The summit of Cangshan Mountain has an altitude of 4122 m, and the other 18 peaks are all above 3500 m. In the valleys between peaks, there are 18 streams originating from the eastern slope of Cangshan Mountain that flow into Erhai Lake, which has a water level of 1964 m and is the 65^th^ largest lake and the 7^th^ largest freshwater lake in China. The forest coverage of Cangshan Mountain is above 90%, and the stream reaches flowing through the mountain are difficult for humans to access and are still pristine. However, when the streams flow out of the mountain, they flow through a narrow plain (around 5 km wide) between Cangshan Mountain and Erhai Lake (Fig. 1). The plain has been intensely exploited for agriculture and urban development. In this study, we sampled five streams. In each stream, the upstream site is located at the foot of Cangshan Mountain, where the stream flows outward from the mountain and is not influenced by anthropogenic activities. The downstream site is located at the mouth of the stream, where the stream flows into the lake and is severely influenced by agriculture and urbanization on the plain.

### Sampling and chemical analyses

Samples were collected in August 2016. At each sample site, we randomly sampled 6–9 submerged rocks from the river cross-section. The benthic biofilm was removed by rigorously brushing an area of 4.5 cm in diameter from the upper surface of each stone using a sterilized nylon brush (changed between samples) and rinsing the slurry with sterile water. For microbial analysis, 10 mL of mixed slurry was filtered through 0.2 μm membrane filters and immediately frozen in liquid nitrogen in the field and transported to the lab for DNA extraction and subsequent analyses. For the biofilm chemical analysis, 20 mL of mixed slurry was filtered through 0.2 μm GF/F filters that were dried at 60 °C. Biofilm carbon (Bio-C) and biofilm nitrogen (Bio-N) was analyzed using an element analyzer (LECO 628). Biofilm phosphorus (Bio-P) was analyzed spectrophotometrically by ascorbic acid colorimetry following digestion with potassium persulfate (EPA 365.3). At each sample site, water samples were collected for nitrogen and phosphorus analysis. Total nitrogen (TN) was analyzed by ion chromatography after persulfate oxidation (EPA 300.0). Nitrate (NO_3_^−^) was determined by ion chromatography (EPA 300.0). Total phosphorus (TP) was analyzed using the ammonium molybdate method after oxidation (EPA 365.3). Soluble reactive phosphorus (SRP) was analyzed using the ammonium molybdate method (EPA 365.3). Ammonium (NH_4_^+^) was analyzed using the indophenol colorimetric method (EPA 350.1). Dissolved organic carbon (DOC) was analyzed on a TOC analyzer (TOC-VCPH; Shimadzu Scientific Instruments, Columbia, Maryland).

### DNA extraction, PCR, and sequencing

Genomic DNA was extracted using the PowerSoil DNA Isolation Kit (MoBio, Carlsbad, CA, USA) following the manufacturer’s protocols. The V3–V4 regions of the 16 S rRNA gene were amplified using 338F-ACTCCTACGGGAGGCAGCA and 806R-GGACTACHVGGGTWTCTAAT (Invitrogen, Vienna, Austria). PCR was performed with a model 2720 thermal cycler (ABI, USA) using the following program: 1-min hot start at 80 °C, 94 °C for 5 min followed by 30 cycles of denaturation at 94 °C for 30 s, followed by annealing at 52 °C for 30 s and 72 °C for 1 min 30 s, with a final extension step at 72 °C for 10 min. Amplified DNA was verified by electrophoresis of the PCR mixtures in 1.0% agarose in 1X TAE buffer and purified using the Gel Extraction Kit (Qiagen, Hilden, Germany). Samples were sent for sequencing on a MiSeq platform.

### Analysis

Bacterial 16 S rRNA sequence data were cleaned using the software package QIIME^40^ and then clustered into operational taxonomic units (OTUs) with a complete linkage algorithm at a 97% sequence identity level. The metagenomes were predicted from 16 S data using PICRUSt^41^. The functional genes associated with nitrogen and carbon metabolism were identified from KEGG (Kyoto Encyclopedia of Genes and Genomes) database^42^. Redundancy analysis (RDA) was conducted to reveal the association of the microbial communities in relation to nutrient factors based on OTU and functional gene abundances (Vegan package 2.4 in R 3.3.2). Correlation analysis was conducted to assess the relationships between the nutrient factors and nitrogen cycle pathways, and the P-value was adjusted with a Bonferroni correction (psych package 1.7.5 in R 3.3.2). PERMANOVA was used to test whether upstream and downstream sites harbor significantly different microbial communities or metagenomes (using PAST 3.0). Mantel tests were run to assess correlations between functional and taxonomic community dissimilarity matrices based on Bray-Curtis distance. Non-metric multidimensional scaling (NMDS) was applied to reveal differences in community composition between microbial assemblages in upstream and downstream sites (using R 3.3.2 and Vegan package 2.4–1). For network analysis, the relative abundances of the OTUs in each sample were used to construct matrices for visualizing interactions between OTUs in the networks (microbial networks of upstream and downstream sites). A Spearman correlation coefficient R score and a P-value were calculated pairwise between the OTUs (the1000 most abundant OTUs) using the Hmisc package (version 4.0–1) in R (version 3.3.2). Only strong (Spearman’s correlation coefficient R > 0.95 or R < −0.95) and strongly significant (P < 0.01) correlations were considered. These correlations were visualized using Cytoscape (version 3.5.0). Each node represents an OTU, and each edge represents a strong and significant correlation. To describe the network topology, a set of node/edge metrics was analyzed using the Network Analyzer plugin within Cytoscape^43^. The modular structures of the networks were analyzed using the ClusterMaker in Cytoscape. Modularity values > 0.4 suggests that the network is modular^44^.

## Results

### Nutrient changes in stream water and benthic biofilms

In the stream water, downstream sites had significantly higher concentrations of TN and NO_3_^−^ than upstream sites (Table 1, t-test, P < 0.05), and the nitrogen was mainly NO_3_^−^ in both the upstream and downstream sites. Moreover, downstream sites had higher concentrations of TP, SRP, and DOC than upstream sites. Accordingly, downstream sites had significantly higher C, N and P contents in the benthic biofilms (Bio-C, Bio-N and Bio-P) than upstream sites (Table 1, t-test, P < 0.05). Bio-N was positively correlated with TN, NO_3_^−^, and TP (Table 2, P < 0.05). Bio-P was positively correlated with TP and SRP but negatively correlated with DOC (Table 2).

**Table 1:** Nutrient variations in stream water and benthic biofilms of upstream sites and downstream sites (Means ± SD, abbreviations as in text).

	Upstream	Downstream	P (t-test)
TN (μg/L)	268.6 ± 65.61	900.0 ± 376.41	0.006
NO_3_^−^ (μg/L)	208.0 ± 59.01	689.8 ± 413.12	0.033
NH_4_^+^ (μg/L)	14.6 ± 6.50	16.0 ± 4.64	0.705
TP (μg/L)	36.4 ± 8.99	50.8 ± 11.73	0.061
SRP (μg/L)	21.8 ± 8.70	31.6 ± 7.06	0.086
DOC (mg/L)	1.1 ± 0.36	1.6 ± 0.38	0.101
Bio-C (nmol/mm^2^)	50.52 ± 28.82	152.02 ± 93.38	0.049
Bio-N (nmol/mm^2^)	1.6 ± 1.35	8.5 ± 6.04	0.037
Bio-P (nmol/mm^2^)	0.45 ± 0.18	1.42 ± 0.85	0.036

**Table 2:** Correlations between nutrient concentrations in stream water and benthic biofilms (abbreviations as in text).

	TN	NO_3_^−^	NH_4_^+^	TP	SRP	DOC
Bio-C	−0.465	−0.486	−0.224	−0.153	−0.049	−0.375
Bio-N	0.854**	0.712*	0.211	0.651*	0.625	−0.383
Bio-P	0.582	0.345	0.168	0.856**	0.790**	−0.722*

Note: “*” indicates level of statistical significance at P < 0.05, “**” indicates P < 0.01.

### Microbial community variation-taxonomic and functional

**Figure 2:**
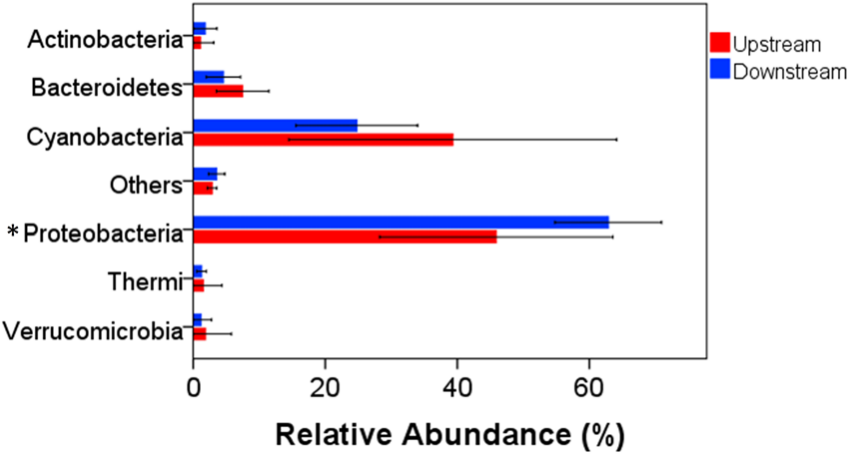
Relative abundance of microorganisms at the phylum level in stream biofilms. Only the phyla with a relative abundance >1% are shown. “Others” represent the unsigned OTUs and the phyla with a relative abundance <1%. Only the relative abundance of proteobacteria was significant between the upstream and downstream sites (“*” indicates level of statistical significance at P < 0.05).

**Figure 3:**
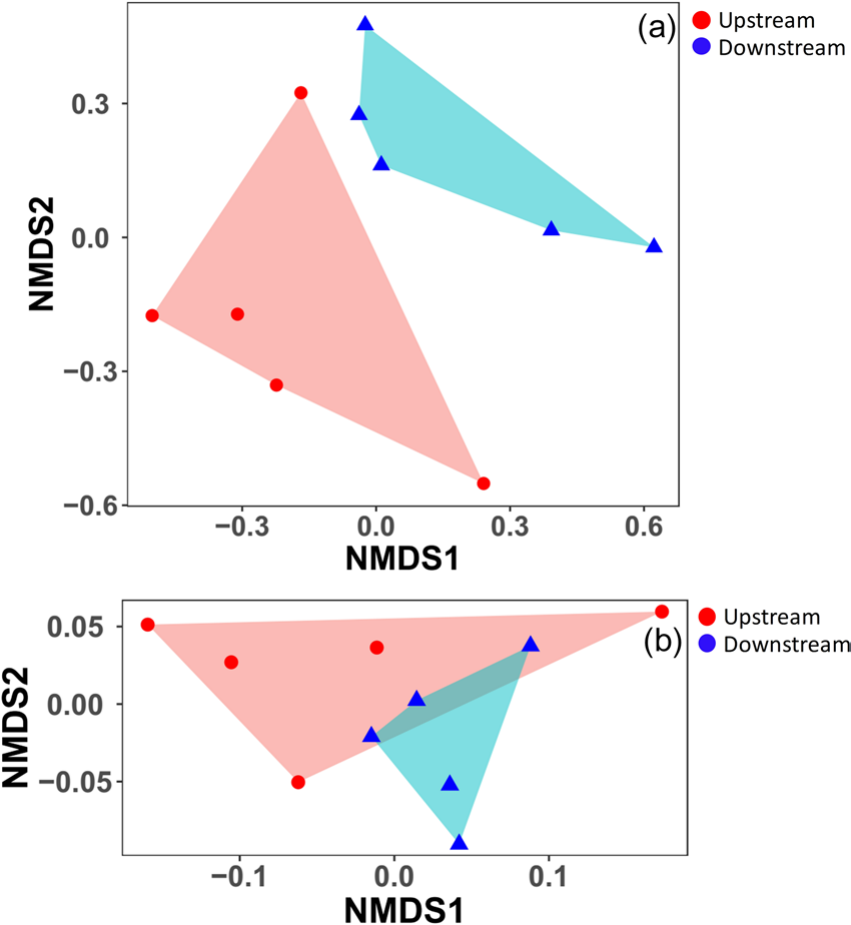
Non-metric multidimensional scaling (NMDS) analysis of the dissimilarities among microbial communities using the Bray-Curtis distances. (**a**) Bray-Curtis distances were calculated using the relative abundance of OTUs. (**b**) Bray-Curtis distances were calculated using the relative abundance of the functional genes predicted by PICRUSt.

**Figure 4:**
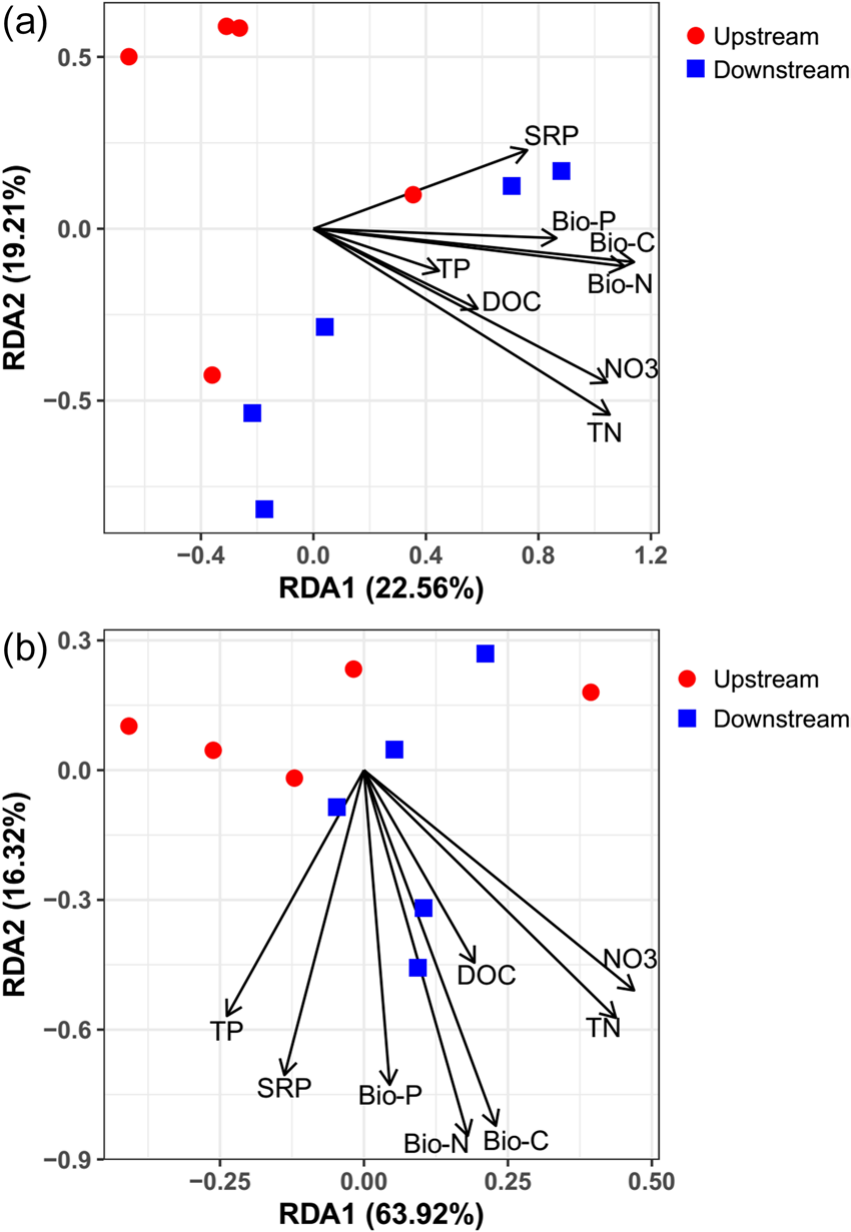
Redundancy analysis (RDA) reveals the association of microbial communities in relation to nutrient factors based on relative abundances of (**a**) OTUs and (**b**) functional genes. Abbreviations as in the text.

After quality filtering, the valid sequences obtained were assigned to 7869 OTUs. The proportion of detected OTUs that overlapped between upstream and downstream was 57.15%. The proportions of detected OTUs that were unique to upstream and downstream sites were 21.17% and 21.69%, respectively. All the OTUs were associated with 28 phyla. The dominant phyla in upstream sites were Proteobacteria (relative abundance of 45.9%) followed by Cyanobacteria (39.3%) and Bacteroidetes (7.5%) (Fig. 2). The dominant phyla in downstream sites were Proteobacteria (62.9%) and Cyanobacteria (24.8%) (Fig. 2). Downstream sites had significantly higher abundances of Proteobacteria than upstream sites (Fig. 2, t-test, P < 0.05), while upstream sites had a higher abundance of Cyanobacteria than downstream sites. NMDS analysis showed that the upstream and downstream sites had different taxonomic communities in the benthic biofilms (Fig. 3a). The Bray-Curtis distance based dissimilarity tests confirmed that the differences were significant (PERMANOVA, F = 1.630, P = 0.028). RDA indicated that TN, NO_3_^−^, Bio-C, and Bio-N were the most significant nutrient factors associated with taxonomic composition (Monte Carlo test, P < 0.05, Fig. 4a). The first two axes explained 41.77% of the taxonomic information (RDA 1: 22.56%; RDA 2: 19.21%).

From the PICRUSt-predicted metagenome results, 5994 functional genes were detected. NMDS analysis showed that the upstream and downstream sites had different functional gene compositions in their benthic biofilms (Fig. 3b). However, the Bray-Curtis distance-based dissimilarity tests showed that the differences were nonsignificant (PERMANOVA, F = 2.203, P = 0.104). RDA analysis indicated that TN, NO_3_^−^, SRP, Bio-C, Bio-N, and Bio-P showed significant relationships with the functional gene composition of the biofilm communities (Monte Carlo test, P < 0.05, Fig. 4b). The first two axes explained 80.24% of the metagenomic variance (RDA 1: 63.92%; RDA 2: 16.32%).

**Figure 5:**
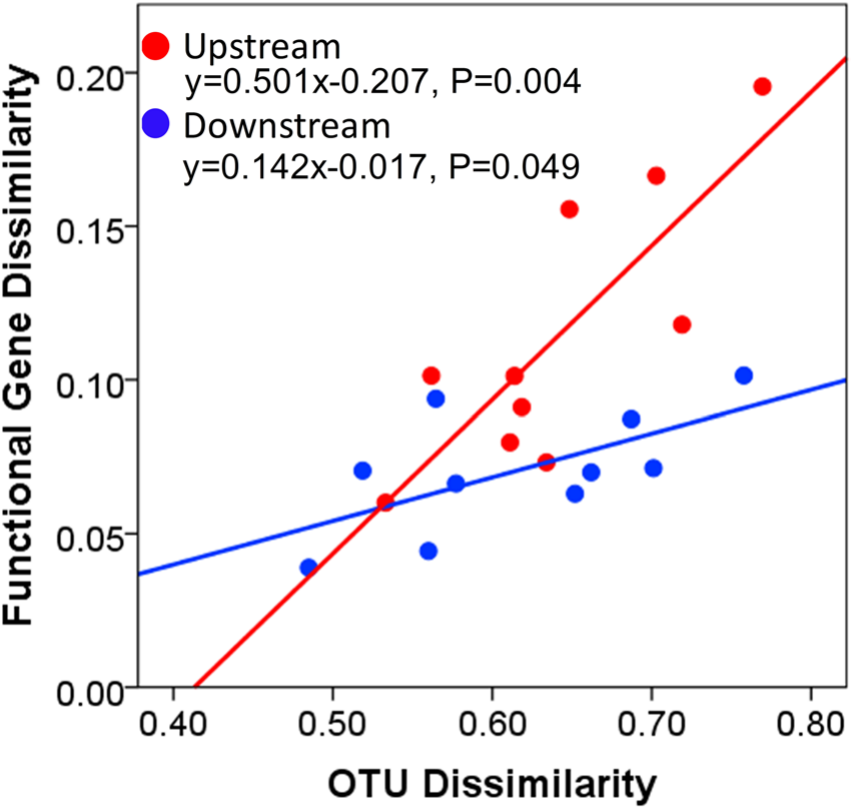
Plots of Mantel tests showing the relationships between the functional gene and OTU dissimilarities. One dot represents one sample pair. Blue dots and line denote downstream sites. Red dots and line denote upstream sites.

To evaluate whether taxonomic differences between microbial communities affect their functional potential, we conducted Mantel tests to analyses the relationships between Bray-Curtis dissimilarities based on the taxon abundances and the functional gene abundances of microbial communities in stream biofilms. Mantel correlation tests revealed significantly positive correlations between the functional and taxonomic dissimilarities in upstream biofilms (Fig. 5, R^2^ = 0.659, P = 0.004) and downstream biofilms (Fig. 5, R^2^ = 0.399, P = 0.049). Moreover, the slope of the linear relationships was smaller in the downstream sites than in the upstream sites.

### Bacteria co-occurrence

**Figure 6:**
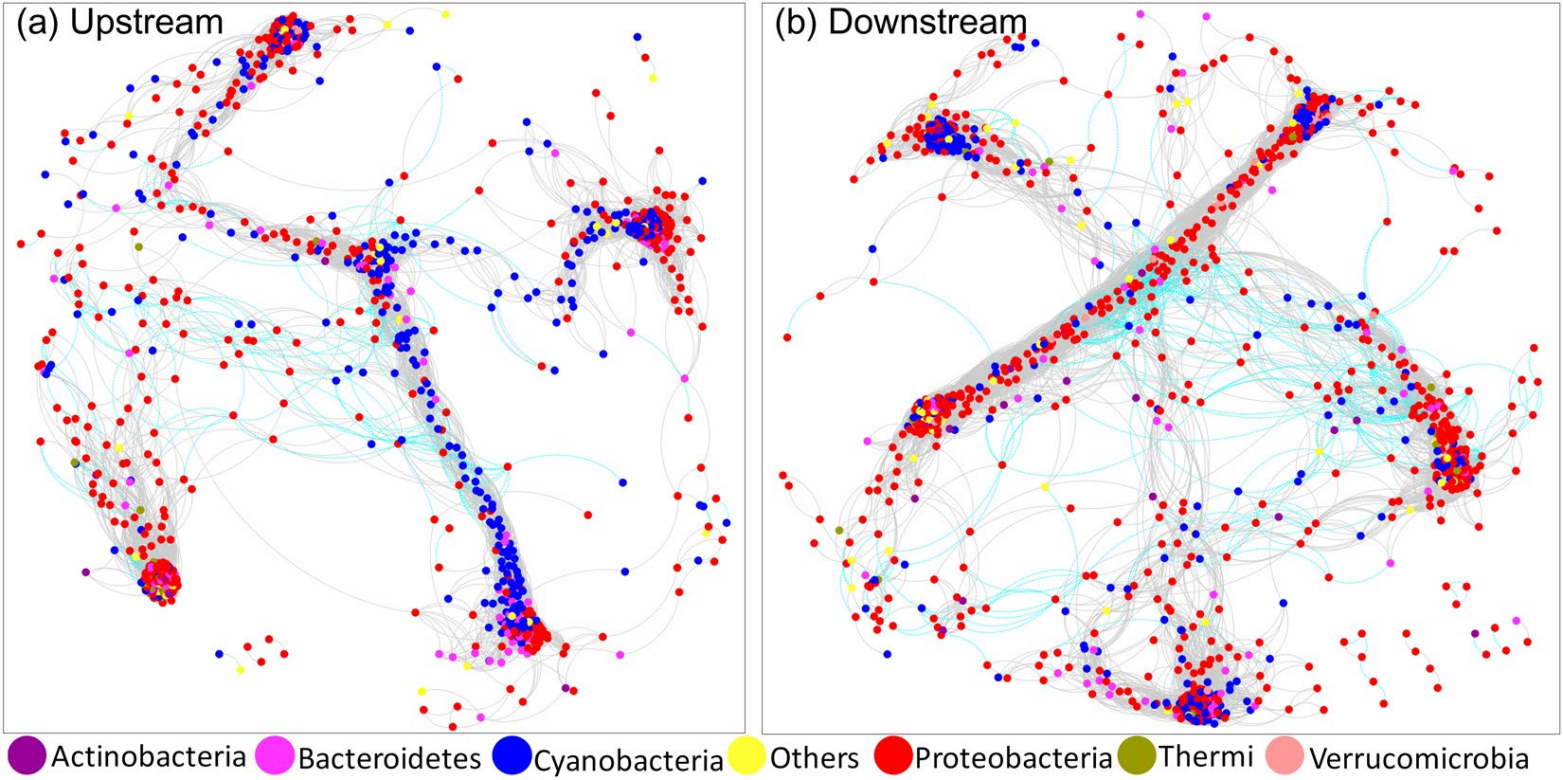
The co-occurrence network of bacterial communities in (**a**) upstream sites and (**b**) downstream sites. Edges represent correlation relationships. The gray solid line indicates positive associations and the cyan dashed line indicate negative associations. Only strong and significant relationships (Spearman R > 0.95 or R < −0.95, P < 0.01) are shown. Circle nodes represent the 1000 most abundant OTUs in upstream and downstream samples. The different colors of the nodes represent bacterial phyla.

We compared the co-occurrence networks of upstream and downstream microbial communities (Fig. 6). The interactions between OTUs (the top 1000 OTUs) in the upstream and downstream sites were described by the topological parameters of the networks (Table 3). In comparing the topological parameters of these two networks (Table 3), the upstream microbial network exhibited a greater values of network diameter, network centralization, network heterogeneity, and characteristic path length. Both networks had a strongly clustered topology, but the upstream microbial network had a lower modularity than the downstream microbial network (Table 3).

**Table 3:** Topological parameters of microbial networks in the upstream and downstream sites.

Topological Parameter	Upstream	Downstream
Network Diameter	20	19
Network Centralization	0.107	0.058
Network Heterogeneity	0.831	0.692
Characteristic Path Length	7.222	6.075
Clustering Coefficient	0.745	0.693
Modularity	0.693	0.779

### Potential metabolisms

**Figure 7:**
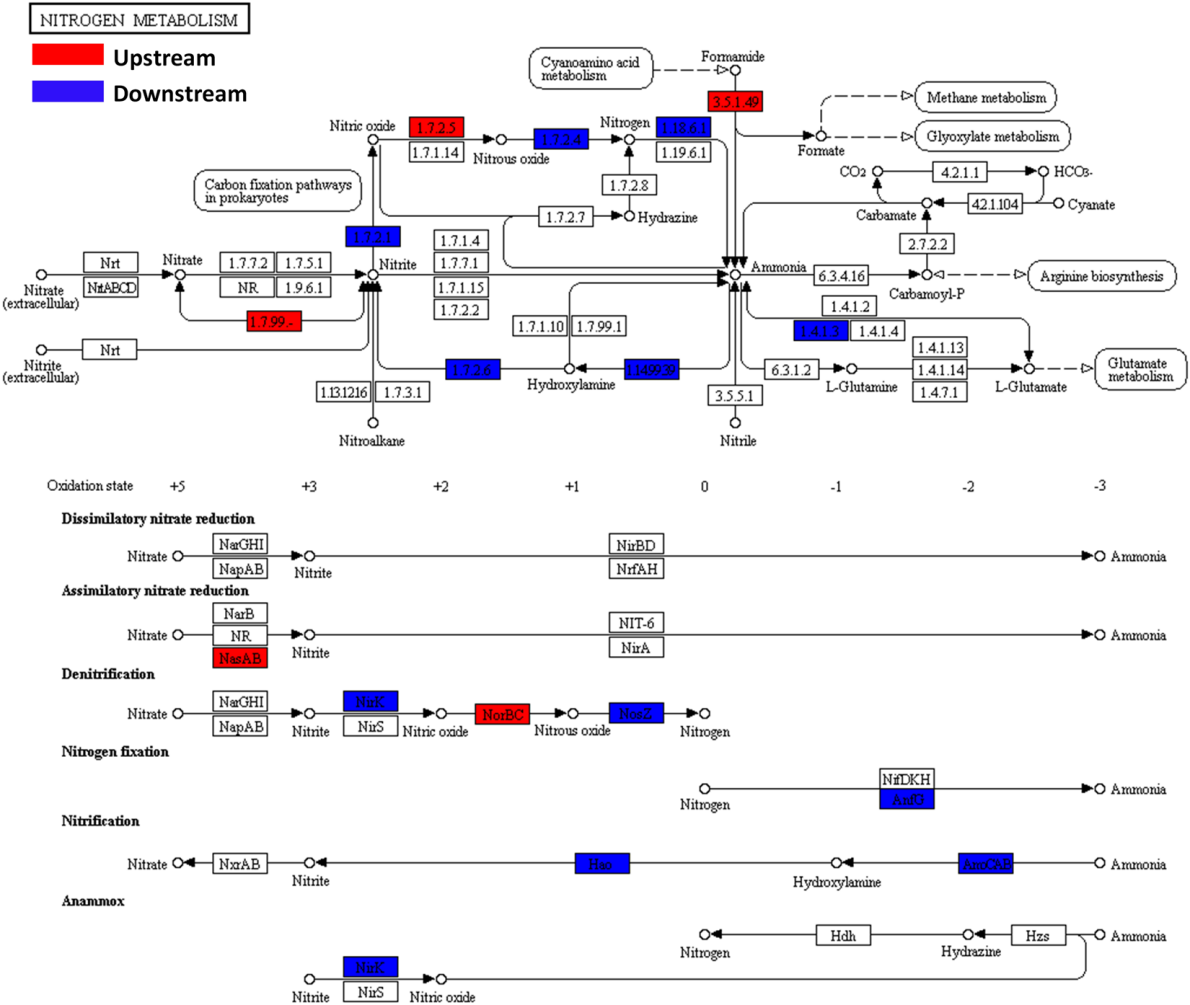
Nitrogen metabolic pathways (map00910) performed by KEGG mapper (http://www.genome.jp/kegg/mapper)^42^. The red box indicates the enzyme that had a significantly high relative abundance in upstream sites. The blue box indicates the enzyme that had a significant high relative abundance in downstream sites.

**Figure 8:**
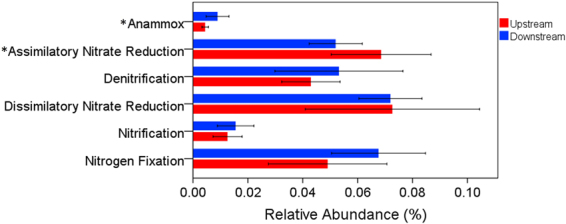
Relative abundances of functional genes encoding the enzymes that catalyze nitrogen cycling pathways. Only the relative abundances of anammox and assimilatory nitrate reduction were significantly different between upstream and downstream (“*” indicates level of statistical significance at P < 0.05).

The N cycle involves four reduction pathways (assimilatory nitrate reduction, dissimilatory nitrate reduction to ammonia, denitrification, and nitrogen fixation) and two oxidation pathways (nitrification and anammox) (Fig. 7). Compared to upstream sites, downstream sites had higher relative abundances of some genes which encoding enzymes associated with denitrification, nitrification, and anammox (Figs 7 and 8), suggesting that downstream sites had higher nitrogen metabolic pathways associated with nitrogen removal. Downstream sites also had a higher relative abundance of nitrogen fixation (Figs 7 and 8). Upstream sites had a higher relative abundance of assimilatory nitrate reduction than downstream sites (Figs 7 and 8). Both upstream and downstream sites had a high relative abundance of dissimilatory nitrate reduction (Fig. 8). Correlation analyses (Table 4) showed that anammox was positively correlated with TN, NO_3_^−^, Bio-C, Bio-N and Bio-P. Assimilatory nitrate reduction was negatively correlated with TN, NO_3_^−^, and Bio-C. Denitrification was positively correlated with Bio-C, Bio-N, and Bio-P. Dissimilatory nitrate reduction was negatively correlated with NH_4_^+^. Nitrification was positively correlated with Bio-C and Bio-P.

**Table 4:** Relationships between the relative abundance of functional genes in benthic biofilms associated with nitrogen cycle pathways and nutrients in stream water and benthic biofilms (abbreviations as in text).

	TN	NO_3_^−^	NH_4_^+^	TP	SRP	DOC	Bio-C	Bio-N	Bio-P
Anammox	0.835**	0.772**	0.288	0.562	0.625	0.494	0.892**	0.930**	0.705*
Assimilatory Nitrate Reduction	−0.678*	−0.647*	0.185	−0.155	−0.245	−0.393	−0.645*	−0.603	−0.454
Denitrification	0.422	0.357	0.309	0.505	0.519	0.173	0.831**	0.782**	0.695*
Dissimilatory Nitrate Reduction	0.003	0.033	−0.735*	−0.520	−0.453	−0.082	−0.206	−0.248	−0.257
Nitrification	0.253	0.166	0.157	0.430	0.377	0.059	0.729*	0.628	0.665*
Nitrogen Fixation	0.295	0.198	0.048	0.302	0.086	0.409	0.630	0.544	0.416

Note: “*” indicates level of statistical significance at P < 0.05, “**” indicates P < 0.01.

**Figure 9:**
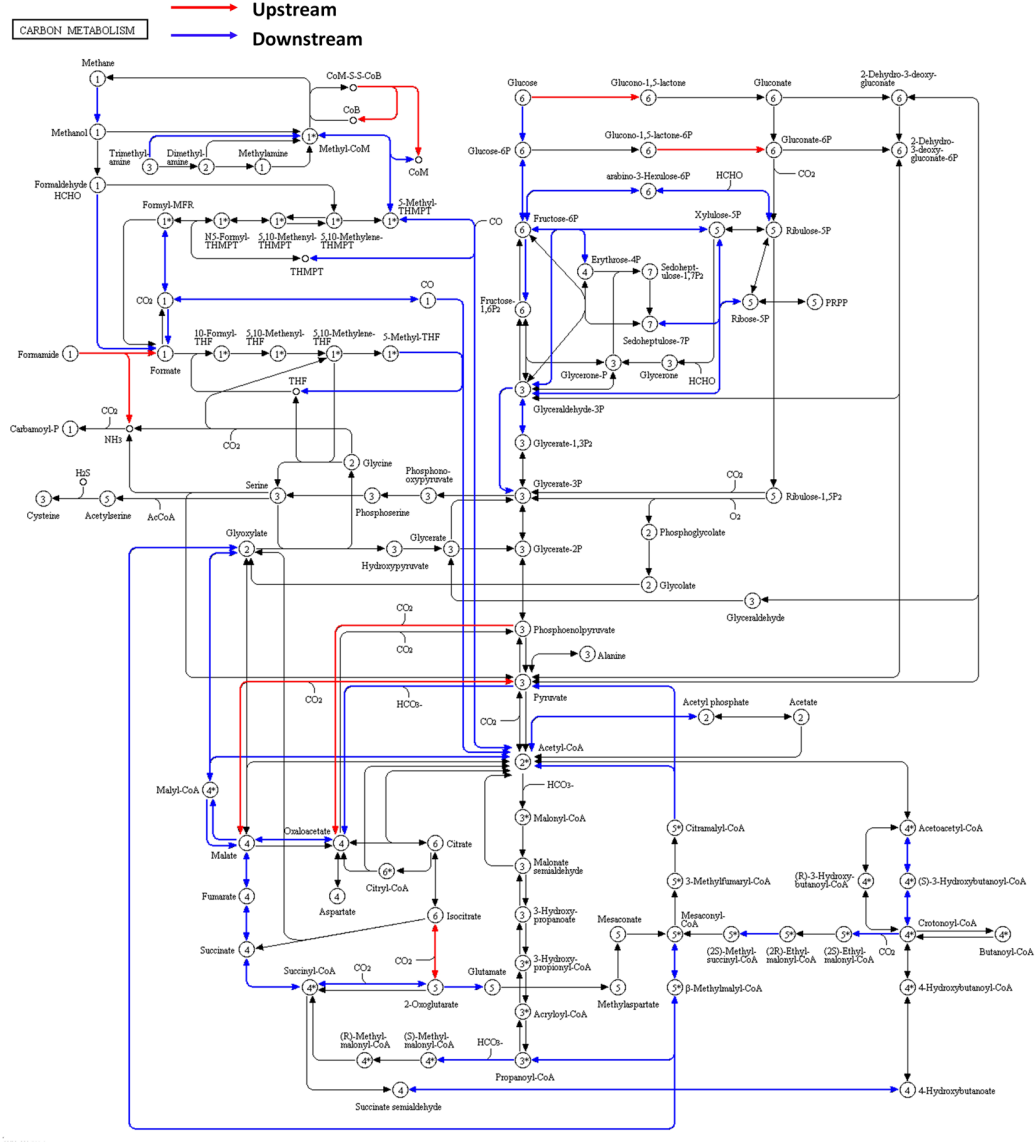
Carbon metabolism pathways (map01200) performed by KEGG mapper (http://www.genome.jp/kegg/mapper)^42^. The red line indicates the pathway that had significantly high relative abundance in upstream sites. The blue line indicates the pathway that had significantly high relative abundance in downstream sites.

For the carbon metabolism, we detected 234 functional genes encoding the core carbon metabolism pathways (Fig. 9). There were 44 functional genes that had higher relative abundances in microbial assemblages in downstream sites than in upstream sites, while only 7 functional genes had higher relative abundances in upstream then in downstream microbial assemblages. This result suggested that microbial assemblages in benthic biofilms had a stronger carbon metabolism in downstream sites than in upstream sites (Fig. 9).

## Discussion

### Anthropogenic impacts on nutrients and biofilm microbial communities

Land use significantly controls the export of nutrient and organic matter from catchments to aquatic ecosystems^11–13^. Agriculture and urban use increase NO_3_^−^, NH_4_^+^, and SRP concentrations as a result of excess fertilizer application^45^, as well as sewage and septic inputs^46,47^. Increased nutrient loading can lead to reduced water quality^48^ and altered stream communities^49,50^. Biofilm nitrogen and phosphorus contents increase with increasing nutrient availability in streams^51,52^. In our study, downstream sites had higher nutrient concentrations in both stream water and benthic biofilms than upstream sites. The nutrient contents in benthic biofilms were closely related to stream-water nutrient concentrations. N and P availability may limit the growth of both biofilm autotrophs and heterotrophs^53,54^. It has been demonstrated that the community composition, diversity, biomass, and stoichiometry of biofilms change significantly with available nutrients in streams^23,28,55,56^. The composition of microbial communities can change in streams influenced by catchment land-use features^57–60^. In our study, downstream sites had a high abundance of proteobacteria while upstream sites had a high abundance of cyanobacteria. The differences in microbial communities were closely correlated with nutrients in both the stream water and biofilms.

Different biomes harbor distinct microbial assemblages^61–63^. It has been demonstrated that functional beta diversity is strongly correlated with taxonomic beta diversity across soil microbial communities^61^. In our study, both upstream and downstream sites had significant linear relationships between OTU dissimilarities and functional gene dissimilarities. The high linear regression slope suggested that the microorganisms in upstream sites had more distinct functional genes than in downstream sites. The microorganisms in downstream sites share more functions than upstream sites, indicating high functional gene redundancy. This also indicated that intense anthropogenic impacts can cause the reduction of functional diversity^64–66^.

Co-occurrence patterns of organisms have been evaluated to reveal community assembly rules and interaction networks in highly complex systems^67^. Network topology (the node distributions and interactions) can affect the stability of the system^38,68^. In previous macroecological studies, communities with tightly connected species were shown to be more susceptible to disturbance^69,70^. The highly connected microbial network of microorganisms in upstream sites suggested that microorganisms have stronger co-occurrence relationships in upstream sites than in downstream sites. A minor disturbance might cause large impacts on upstream network because of the complex relationships among OTUs. Furthermore, the microbial assemblages in both upstream and downstream sites exhibited a modular structure (Table 3, modularity values > 0.4 suggest that the network is modular, Newman 2006). In a biotic network, highly interconnected species are grouped into a module. In a module, species interactions are more frequent and intensive than in the rest of the community^38,44^. Niche differentiation can lead to higher modularity in a community^38^. In our study, the high modularity of both upstream and downstream microbial networks suggested that the upstream and downstream sites of the stream had heterogeneous habitats offering rich niches for microorganisms, especially in downstream sites which receive more nutrient and organic matter from the catchment.

### Anthropogenic influences on biofilm metabolic potentials

Microorganisms in biofilms are major components of the biogeochemical cycles in stream ecosystems^71^. Human activities modify land use and affect quantity and quality of nutrient and organic matter in streams^3,72^. As a result of anthropogenic impacts on catchments, changes in the community composition of benthic biofilms result in the alteration of stream ecosystem functions^6,73^.

Nitrogen has various chemical forms and is cycled by a suite of biogeochemical processes^74^, which are catalyzed by various microbe-excreted enzymes^75,76^. The assimilatory pathways require energy and start with the reduction of NO_3_^−^ to NO_2_^−^ and then to NH_4_^+^, which is highly bioavailable and can be readily used by the cell for the synthesis of amino acids and nucleotides or can be transformed by nitrification or anammox^77^. Dissimilatory nitrate reduction to ammonia uses N-compounds to provide energy (ATP) to microbes and is another catabolic pathway that can retain the nitrogen in the system in a bioavailable form (NH_4_^+^) for further biological processes^78,79^. Nitrogen fixation is an energetically demanding process requiring 16 molecules of ATP to break the triple-N bond. Only a few organisms, including cyanobacteria and certain other genera, can carry out this process^80,81^. Low NH_4_^+^ can stimulate some N_2_-fixing bacteria^82–84^. Nitrification is an essential process in the nitrogen cycle performed by nitrifiers converting ammonium to nitrate. As a competing process to nitrate reduction pathways, denitrification is the main biological process for the removal of N from freshwater systems^79,85^. Anammox plays an important role in the nitrogen cycle, turning nitrite and ammonia to dinitrogen^86^, and is another microbial process that releases fixed nitrogen from the environment as dinitrogen^87–90^. In our study, a higher relative abundance of denitrification, nitrification, and anammox suggested that nitrogen removal was stronger in downstream sites than in upstream sites.

Furthermore, nitrogen metabolic pathways are regulated by environmental redox states^91,92^, nutrient availability, and other abiotic factors. High concentrations of NO_3_^−^ can stimulate microorganisms to increase populations containing the functional genes associated with nitrate reduction. Anammox is influenced by NO_3_^−^ concentration, NH_4_^+^ concentration, C/N ratio, and pH^93^. Low NH_4_^+^ can stimulate functional genes associated with nitrogen fixation^82–84^. Nitrification can be affected by temperature, salinity, light, organic matter concentration, substrate concentrations, pH, and oxygen concentration^94^. Our study showed that anammox was positively correlated with TN, NO_3_^−^, Bio-C, Bio-N and Bio-P. Assimilatory nitrate reduction was negatively correlated with TN, NO_3_^−^, and Bio-C. Dissimilatory nitrate reduction was negatively correlated with NH_4_^+^. Denitrification was positively correlated with Bio-C, Bio-N and Bio-P. Nitrification was positively correlated with Bio-C and Bio-P (Table 4). These results suggest that human land use significantly increases nutrient and organic carbon in stream water and biofilms, and the increased nutrient had severe impacts on the nitrogen cycle in streams.

In stream ecosystems, the processing of organic matter is another major function that bacterial communities mediate^29^. Carbon transported by streams provides a major source of energy for aquatic food webs and is a significant component of the global carbon cycle^31,32,95^. Anthropogenic land use contributed significantly to accelerated transport of carbon from terrestrial environments to aquatic ecosystems^31,96–98^. On the other hand, anthropogenic land use can accelerate the transition of streams from transporters to transformers of carbon^31^. In stream ecosystems, a substantial portion of the organic matter are derived from terrestrial environments and serve as a substantial resource fueling riverine bacterial communities^29^. Small streams can mineralize large quantities of the organic carbon load because of the bacterial carbon metabolism, which results in increased losses of CO_2_ to the atmosphere and with implications for ecosystem services^8,34,99^. Moreover, the increased nutrient availability can further stimulate whole-stream metabolism of terrestrial carbon^8,100^. In our study, the overall carbon metabolism of the benthic biofilms was enhanced by the intense anthropogenic activities.

## Conclusions

Anthropogenic activities have caused the increase of nitrogen and carbon loads in streams and rivers. A portion of increased nitrogen and carbon loads are exported to downstream lake and coastal waters, while a substantial proportion are also transformed along streams and rivers^35,101^. Furthermore, anthropogenic land use accelerates the transition of streams from transporters to transformers of nutrient and organic carbon^31^. In our study, anthropogenic influenced downstream sites had higher relative abundances of functional gene encoding nitrogen and carbon metabolisms compared to upstream sites with relatively pristine catchments. The results allow us to understand how microbial community structures and metabolism response to influences of anthropogenic land use.

## References

[1] Foley JA (2005). Global consequences of land use. Science..

[2] Williamson CE, Dodds W, Kratz TK, Palmer MA (2008). Lakes and streams as sentinels of environmental change in terrestrial and atmospheric processes. Front. Ecol. Environ..

[3] Deegan LA (2011). Amazon deforestation alters small stream structure, nitrogen biogeochemistry and connectivity to larger rivers. Biogeochemistry..

[4] Figueiredo RO, Markewitz D, Davidson EA, Schuler AE (2010). Dos S. Watrin, O. and de Souza Silva, P. Land-use effects on the chemical attributes of low-order streams in the eastern Amazon. J. Geophys. Res..

[5] Vannote RL, Minshall GW, Cummins KW, Sedell JR, Cushing CE (1980). The river continuum concept. Can. J. Fish. Aquat. Sci..

[6] Burgos-Caraballo S, Cantrell SA, Ramirez A (2014). Diversity of Benthic Biofilms Along a Land Use Gradient in Tropical Headwater Streams, Puerto Rico. Microb. Ecol..

[7] Allan JD (2004). Landscapes and riverscapes: The influence of land use on stream ecosystems. Annu. Rev. Ecol. Evol. S..

[8] Rosemond AD (2015). Experimental nutrient additions accelerate terrestrial carbon loss from stream ecosystems. Science..

[9] Godwin CM, Carrick HJ (2008). Spatio-temporal variation of periphyton biomass and accumulation in a temperate spring-fed stream. Aquat. Ecol..

[10] Larned ST (2010). A prospectus for periphyton: recent and future ecological research. J. N. Am. Benthol. Soc..

[11] Erol A, Randhir TO (2013). Watershed ecosystem modeling of land-use impacts on water quality. Ecol. Model..

[12] Umbanhowar C (2015). Lake-landscape connections at the forest-tundra transition of northern Manitoba. Inland Waters..

[13] Abell JM, Oezkundakci D, Hamilton DP, Miller SD (2011). Relationships between land use and nitrogen and phosphorus in New Zealand lakes. Mar. Freshwater Res..

[14] Teittinen A, Taka M, Ruth O, Soininen J (2015). Variation in stream diatom communities in relation to water quality and catchment variables in a boreal, urbanized region. Sci. Total Environ..

[15] Walker CE, Pan YD (2006). Using diatom assemblages to assess urban stream conditions. Hydrobiologia..

[16] Smucker NJ, Detenbeck NE, Morrison AC (2013). Diatom responses to watershed development and potential moderating effects of near-stream forest and wetland cover. Freshw. Sci..

[17] Ren Z, Jiang ZY, Cai QH (2013). Longitudinal patterns of periphyton biomass in Qinghai–Tibetan Plateau streams: An indicator of pasture degradation?. Quatern. Int..

[18] O’Brien PJ, Wehr JD (2010). Periphyton biomass and ecological stoichiometry in streams within an urban to rural land-use gradient. Hydrobiologia..

[19] Geesey GG, Mutch R, Costerton JT, Green RB (1978). Sessile bacteria: an important component of the microbial population in small mountain streams. Limnol. Oceanogr..

[20] Schiller DV, Marti E, Riera JL, Sabater F (2007). Effects of nutrients and light on periphyton biomass and nitrogen uptake in Mediterranean streams with contrasting land uses. Freshwater Biol..

[21] Battin TJ, Kaplan LA, Newbold JD, Hansen C (2003). Contributions of microbial biofilms to ecosystem processes in stream mesocosms. Nature..

[22] Buchkowski RW, Schmitz OJ, Bradford MA (2015). Microbial stoichiometry overrides biomass as a regulator of soil carbon and nitrogen cycling. Ecology..

[23] Van Horn DJ, Sinsabaugh RL, Takacs-Vesbach CD, Mitchell KR, Dahm CN (2011). Response of heterotrophic stream biofilm communities to a gradient of resources. Aquat. Microb. Ecol..

[24] Peterson BJ (2001). Control of nitrogen export from watersheds by headwater streams. Science..

[25] Burgin AJ, Hamilton SK (2007). Have we overemphasized the role of denitrification in aquatic ecosystems? A review of nitrate removal pathways. Front. Ecol. Environ..

[26] Ishida CK, Arnon S, Peterson CG, Kelly JJ, Gray KA (2008). Influence of Algal Community Structure on Denitrification Rates in Periphyton Cultivated on Artificial Substrata. Microb. Ecol..

[27] Mulholland PJ (2008). Stream denitrification across biomes and its response to anthropogenic nitrate loading. Nature..

[28] Drake WM (2012). The effect of periphyton stoichiometry and light on biological phosphorus immobilization and release in streams. Limnology..

[29] Becker JC, Rodibaugh KJ, Hahn D, Nowlin WH (2017). Bacterial community composition and carbon metabolism in a subtropical riverscape. Hydrobiologia..

[30] Cole JJ (2007). Plumbing the global carbon cycle: integrating inland waters into the terrestrial carbon budget. Ecosystems..

[31] Smith RM, Kaushal SS (2015). Carbon cycle of an urban watershed: exports, sources, and metabolism. Biogeochemistry..

[32] Battin TJ (2008). Biophysical controls on organic carbon fluxes in fluvial networks. Nat. Geosci..

[33] Raymond PA (2013). Global carbon dioxide emissions from inland waters. Nature..

[34] Hall RO, Tank JL, Baker MA, Rosi-Marshall EJ, Hotchkiss ER (2016). Metabolism, Gas Exchange, and Carbon Spiraling in Rivers. Ecosystems..

[35] Kaushal SS (2014). Longitudinal patterns in carbon and nitrogen fluxes and stream metabolism along an urban watershed continuum. Biogeochemistry..

[36] Li XY, Ma YJ, Xu HY, Wang JH, Zhang DS (2009). Impact of land use and land cover change on environmental degradation in Lake Qinghai watershed, northeast Qinghai-Tibet Plateau. Land Degrad. Dev..

[37] Cronan CS (2012). Biogeochemistry of the Penobscot River watershed, Maine, USA: nutrient export patterns for carbon, nitrogen, and phosphorus. Environ. Monit. Assess..

[38] Freedman ZB, Zak DR (2015). Atmospheric N deposition alters connectance, but not functional potential among saprotrophic bacterial communities. Mol. Ecol..

[39] Wang, K. et al. Regional variations in the diversity and predicted metabolic potential of benthic prokaryotes in coastal northern Zhejiang, East China Sea. *Sci. Rep.-UK*. **6**, (2016). 10.1038/srep38709 PMC5137025 27917954

[40] Caporaso JG (2010). QIIME allows analysis of high-throughput community sequencing data. Nat. Methods..

[41] Langille MGI (2013). Predictive functional profiling of microbial communities using 16S rRNA marker gene sequences. Nat. Biotechnol..

[42] Kanehisa M, Goto S (2000). KEGG: kyoto encyclopedia of genes and genomes. Nucleic Acids Res..

[43] Assenov Y, Ramirez F, Schelhorn S, Lengauer T, Albrecht M (2008). Computing topological parameters of biological networks. Bioinformatics..

[44] Newman ME (2006). Modularity and community structure in networks. P. Natl. Acad. Sci. Usa..

[45] Royer TV, Tank JL, David MB (2004). Transport and fate of nitrate in headwater agricultural streams in Illinois. J. Environ. Qual..

[46] Walsh CJ (2005). The urban stream syndrome: current knowledge and the search for a cure. J. N. Am. Benthol. Soc..

[47] Paul MJ, Meyer JL (2001). Streams in the urban landscape. Annual review of Ecology and Systematics..

[48] Dodds WK, Welch EB (2000). Establishing nutrient criteria in streams. J. N. Am. Benthol. Soc..

[49] Kohler TJ (2012). Flow, nutrients, and light availability influence Neotropical epilithon biomass and stoichiometry. Freshw. Sci..

[50] Justus BG, Petersen JC, Femmer SR, Davis JV, Wallace JE (2010). A comparison of algal, macroinvertebrate, and fish assemblage indices for assessing low-level nutrient enrichment in wadeable Ozark streams. Ecol. Indic..

[51] Frost PC, Cross WF, Benstead JP (2005). Ecological stoichiometry in freshwater benthic ecosystems: an introduction. Freshwater Biol..

[52] Kohler TJ, Murdock JN, Gido KB, Dodds WK (2011). Nutrient loading and grazing by the minnow Phoxinus erythrogaster shift periphyton abundance and stoichiometry in mesocosms. Freshwater Biol..

[53] Tank JL, Dodds WK (2003). Nutrient limitation of epilithic and epixylic biofilms in ten North American streams. Freshwater Biol..

[54] Johnson LT, Tank JL, Dodds WK (2009). The influence of land use on stream biofilm nutrient limitation across eight North American ecoregions. Can. J. Fish. Aquat. Sci..

[55] Fanta SE, Hill WR, Smith TB, Roberts BJ (2010). Applying the light: nutrient hypothesis to stream periphyton. Freshwater Biol..

[56] Hill WR, Rinchard J, Czesny S (2011). Light, nutrients and the fatty acid composition of stream periphyton. Freshwater Biol..

[57] Wang SY, Sudduth EB, Wallenstein MD, Wright JP, Bernhardt ES (2011). Watershed urbanization alters the composition and function of stream bacterial communities. PLoS One..

[58] Carrino-Kyker SR, Swanson AK, Burke DJ (2011). Changes in eukaryotic microbial communities of vernal pools along an urban–rural land use gradient. Aquat. Microb. Ecol..

[59] Perryman SE, Rees GN, Walsh CJ (2008). Analysis of denitrifying communities in streams from an urban and non-urban catchment. Aquat. Ecol..

[60] Lear G (2013). The biogeography of stream bacteria. Global Ecol. Biogeogr..

[61] Fierer N (2012). Cross-biome metagenomic analyses of soil microbial communities and their functional attributes. P. Natl. Acad. Sci. Usa..

[62] Hugerth LW (2015). Metagenome-assembled genomes uncover a global brackish microbiome. Genome Biol..

[63] Louca S, Parfrey LW, Doebeli M (2016). Decoupling function and taxonomy in the global ocean microbiome. Science..

[64] Cardinale BJ (2012). Biodiversity loss and its impact on humanity. Nature..

[65] Jung J, Philippot L, Park W (2016). Metagenomic and functional analyses of the consequences of reduction of bacterial diversity on soil functions and bioremediation in diesel-contaminated microcosms. Sci Rep..

[66] Elmqvist T (2003). Response diversity, ecosystem change, and resilience. Front. Ecol. Environ..

[67] Fuhrman JA (2009). Microbial community structure and its functional implications. Nature..

[68] Barberan A, Bates ST, Casamayor EO, Fierer N (2012). Using network analysis to explore co-occurrence patterns in soil microbial communities. Isme J..

[69] Montoya JM, Pimm SL, Sole RV (2006). Ecological networks and their fragility. Nature..

[70] Saavedra S, Stouffer DB, Uzzi B, Bascompte J (2011). Strong contributors to network persistence are the most vulnerable to extinction. Nature..

[71] Battin TJ, Besemer K, Bengtsson MM, Romani AM, Packmann AI (2016). The ecology and biogeochemistry of stream biofilms. Nat. Rev. Microbiol..

[72] Boechat IG, Kruger A, Giani A, Figueredo CC, Gucker B (2011). Agricultural land-use affects the nutritional quality of stream microbial communities. FEMS Microbiol. Ecol..

[73] Allison SD, Martiny JBH (2008). Resistance, resilience, and redundancy in microbial communities. P. Natl. Acad. Sci. Usa..

[74] Ollivier J (2011). Nitrogen turnover in soil and global change. FEMS Microbiol. Ecol..

[75] Falkowski PG, Fenchel T, Delong EF (2008). The microbial engines that drive Earth’s biogeochemical cycles. Science..

[76] Gruber N, Galloway JN (2008). An Earth-system perspective of the global nitrogen cycle. Nature..

[77] Richardson DJ, Watmough NJ (1999). Inorganic nitrogen metabolism in bacteria. Curr. Opin. Chem. Biol..

[78] Zumft WG (1997). Cell biology and molecular basis of denitrification. Microbiol. Mol. Biol. R..

[79] Tiedje JM, Sexstone AJ, Myrold DD, Robinson JA (1983). Denitrification: ecological niches, competition and survival. Antonie van Leeuwenhoek..

[80] Bernhard A (2012). The nitrogen cycle: Processes, players, and human impact. Nature Education Knowledge..

[81] Burris RH, Roberts GP (1993). Biological nitrogen fixation. Annu. Rev. Nutr..

[82] Smith VH, Tilman GD, Nekola JC (1999). Eutrophication: impacts of excess nutrient inputs on freshwater, marine, and terrestrial ecosystems. Environ. Pollut..

[83] Guildford SJ, Hecky RE (2000). Total nitrogen, total phosphorus, and nutrient limitation in lakes and oceans: Is there a common relationship?. Limnol. Oceanogr..

[84] Meeks JC, Wycoff KL, Chapman JS, Enderlin CS (1983). Regulation of expression of nitrate and dinitrogen assimilation by Anabaena species. Appl. Environ. Microb..

[85] Seitzinger SP (1988). Denitrification in freshwater and coastal marine ecosystems: ecological and geochemical significance. Limnol. Oceanogr..

[86] Van Niftrik L, Jetten MSM (2012). Anaerobic Ammonium-Oxidizing Bacteria: Unique Microorganisms with Exceptional Properties. Microbiol. Mol. Biol. R..

[87] Kuypers M (2003). Anaerobic ammonium oxidation by anammox bacteria in the Black Sea. Nature..

[88] Dalsgaard T, Canfield DE, Petersen J, Thamdrup B (2003). and Acuna-Gonzalez. J. N-2 production by the anammox reaction in the anoxic water column of Golfo Dulce, Costa Rica. Nature..

[89] Thamdrup B, Dalsgaard T (2002). Production of N-2 through anaerobic ammonium oxidation coupled to nitrate reduction in marine sediments. Appl. Environ. Microb..

[90] Kartal B (2011). Molecular mechanism of anaerobic ammonium oxidation. Nature..

[91] Rosswall T (1982). Microbiological regulation of the biogeochemical nitrogen cycle. Plant Soil..

[92] Lamba S (2017). Organization of biogeochemical nitrogen pathways with switch-like adjustment in fluctuating soil redox conditions. Royal Society Open Science..

[93] Yang X (2015). Potential Contribution of Anammox to Nitrogen Loss from Paddy Soils in Southern China. Appl. Environ. Microb..

[94] Ward, B. B., Arp, D. J. & Klotz, M. G. Nitrification. American Society for Microbiology Press. (2011).

[95] Cole JJ (2007). Plumbing the Global Carbon Cycle: Integrating Inland Waters into the Terrestrial Carbon Budget. Ecosystems..

[96] Duan S, Amon RM, Brinkmeyer RL (2014). Tracing sources of organic matter in adjacent urban streams having different degrees of channel modification. Sci. Total Environ..

[97] Lu YH (2014). Effects of land use on sources and ages of inorganic and organic carbon in temperate headwater streams. Biogeochemistry..

[98] Tank JL, Rosi-Marshall EJ, Griffiths NA, Entrekin SA, Stephen ML (2010). A review of allochthonous organic matter dynamics and metabolism in streams. J. N. Am. Benthol. Soc..

[99] Marcarelli AM, Baxter CV, Mineau MM, Hall RO (2011). Quantity and quality: unifying food web and ecosystem perspectives on the role of resource subsidies in freshwaters. Ecology..

[100] Roley SS (2014). The influence of floodplain restoration on whole-stream metabolism in an agricultural stream: insights from a 5-year continuous data set. Freshw. Sci..

[101] Galloway JN (2003). The nitrogen cascade. BioScience..

